# Global Context Attention for Robust Visual Tracking

**DOI:** 10.3390/s23052695

**Published:** 2023-03-01

**Authors:** Janghoon Choi

**Affiliations:** Graduate School of Data Science, Kyungpook National University, Daegu 41566, Republic of Korea; jhchoi09@knu.ac.kr

**Keywords:** visual tracking, object tracking, attention models, model-free tracking

## Abstract

Although there have been recent advances in Siamese-network-based visual tracking methods where they show high performance metrics on numerous large-scale visual tracking benchmarks, persistent challenges regarding the distractor objects with similar appearances to the target object still remain. To address these aforementioned issues, we propose a novel global context attention module for visual tracking, where the proposed module can extract and summarize the holistic global scene information to modulate the target embedding for improved discriminability and robustness. Our global context attention module receives a global feature correlation map to elicit the contextual information from a given scene and generates the channel and spatial attention weights to modulate the target embedding to focus on the relevant feature channels and spatial parts of the target object. Our proposed tracking algorithm is tested on large-scale visual tracking datasets, where we show improved performance compared to the baseline tracking algorithm while achieving competitive performance with real-time speed. Additional ablation experiments also validate the effectiveness of the proposed module, where our tracking algorithm shows improvements in various challenging attributes of visual tracking.

## 1. Introduction

Visual tracking is a fundamental field in computer vision research that has seen practical applications in automated surveillance, autonomous driving, and robotics. The problem of visual tracking is formulated as follows: given the initial target bounding box coordinates along with the first frame of a video sequence, the goal of a visual tracking algorithm is to estimate the bounding box coordinates of the target object in the subsequent frames of the video. While performing tracking, there are several challenging circumstances, such as target scale change, illumination change, occlusion from other objects, deformation of the target, and motion blur. A tracking algorithm must successfully identify the target object under these challenging attribute changes. Among many of these attributes, distractor objects of similar appearance to the target object often cause the tracker to drift away from the target object, making the long-term tracking task even more challenging.

Recently, with the wide application of covolutional neural networks (CNN) in various computer vision applications [1,2], Siamese-network-based tracking algorithms gained attention due to their simplicity and fast speed owing to the removal of the online update process. SiamFC [3] was one of the first works to employ this framework, where the Siamese network receives two image patches, a target patch and a search patch, and the network extracts the feature representations from both patches and determines the position of the target patch inside the search patch through a cross-correlation operation. The whole process is performed in a fully convolutional manner and can be trained end-to-end. Many following works focused on improving SiamFC in terms of network architecture design by employing deeper and wider backbone networks [4,5], by adding region proposal networks [6] or centerness estimation for better localization [7,8], and many other approaches to improve its performance [9,10,11].

However, many existing tracking algorithms, including Siamese-network-based trackers, still focus on short-term tracking scenarios [12,13], where they make strong assumptions on the smoothness of the target’s motion. To impose these assumptions, tracking algorithms often assume a small search window around the target’s previous position and penalize large displacements using handcrafted cosine window functions with predesignated sizes and weights. While this strategy is effective for short-term tracking scenarios when the hyperparameters are properly tuned, this may cause the trackers to be susceptible to error accumulation and drift in long-term tracking scenarios [14,15], where the target can move abruptly due to camera motion, distractor objects of similar appearance are more likely to appear in a scene, and the target can show out-of-frame disappearances. To address the aforementioned issues, global search strategy-based tracking algorithms [16,17] were proposed with the benefits of re-detecting the target after long-term disappearances, scene changes, and recovery from occasional drifts caused by distractor objects. Although a global, full-frame search strategy for Siamese-network-based tracking algorithms [16,17] showed improved performance on various long-term tracking scenarios, the issue originating from distractor objects of similar appearance to the target object persists, where the tracking algorithms are often trained to find the single most similar-looking region inside a given frame image without consideration of the global context among numerous objects inside the scene.

To address the issues arising from the lack of context modeling, we propose a novel global context attention module for a Siamese-network-based tracking algorithm, where the tracker can adaptively choose the relevant feature channels and spatial parts of the target object to improve its discriminability inside a given scene based on the aggregated information on the context of objects. For implementation, we improve upon the baseline tracker GlobalTrack [16], which is a two-stage, full-frame search-based tracking algorithm, where we chose this baseline tracker so that the tracker can fully utilize the global context information inside a given scene. GlobalTrack [16] also does not employ any motion model; thus, the benefits of our proposed context attention module can be analyzed more accurately without the influence of the hyperparameter tuning of the motion model. The proposed global context attention module receives the intermediate feature correlation map to extract the contextual information of the scene and generates the channel-wise and spatial attention weights that are utilized to modulate the target feature representation for improved discriminability.

**Figure 1:**
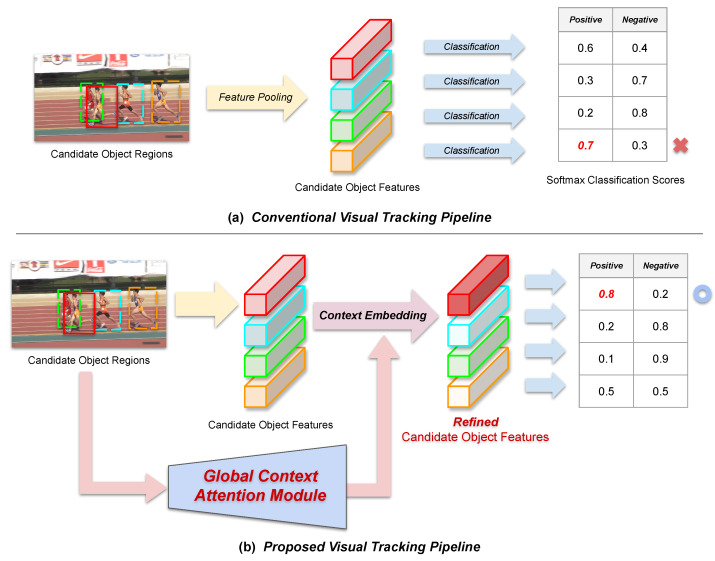
Motivation for the proposed tracking framework.

In order to show the effectiveness of our proposed method, we perform experiments on recent large-scale visual tracking datasets to demonstrate the competitive performance of the proposed algorithm. We also perform ablation experiments to further validate the improvements owing to the proposed global context attention module. Moreover, our framework is designed to run at real-time speeds. Our overall tracking framework is shown in Figure 1. In (a), we show conventional tracking algorithms that treat multiple candidate regions independently, whereas in (b), our tracker learns to embed the global context information into the feature representation. Our proposed tracker employs the global context attention module to model the context of objects in a given scene, where it can provide the baseline tracker with relevant channel and spatial attention weights for improved discriminability of the target object in the feature space.

## 2. Related Work

Online tracking algorithms formulate the visual tracking problem as *tracking-by-detection*, where a tracker attempts to locate the target object inside a search region by solving a binary classification problem. A classifier model is trained to classify the target region as positive and the background region as negative, where the tracker chooses the region with the highest positive score as the output. With the use of powerful feature representation and classifier models based on neural networks, visual tracking algorithms also took advantage of these models. Starting from early CNN-based tracker models using denoising autoencoders [18], MDNet [19] and RT-MDNet [20] used VGG-based [21] feature representation to train a binary classifier *online*. A line of tracking algorithms using correlation filters [22,23,24,25] also utilized CNN-based feature representation, where correlation filters are trained on the feature maps with 2D Gaussian labels to obtain maximum response value at the center of the target object.

Afterwards, the use of a Siamese network also gained traction owing to its simplicity and fully convolutional approach. Starting from SiamFC [3], where a shared CNN feature extractor [1] is used to extract features from both target and search image patches, a subsequent cross-correlation operation between features results in a score map where the position with the maximum score can be chosen for the output. To improve upon SiamFC, there have been numerous approaches where region proposal networks [6] are employed [7,26], deeper and wider feature extractors are used [4,5], a target centerness aware branch is added [8,10], and lightweight networks are used for further speed-up [27,28]. Recently, transformer [29] architecture, which is widely used in natural language and sequence modeling, was also employed into computer vision applications and visual tracking algorithms, especially Siamese-network-based visual tracking algorithms. TransT [30] was one of the first works to employ transformer architecture to substitute the feature cross-correlation layer with its proposed feature fusion block, composed of multiple multi-head self-attention modules. Other approaches include using spatiotemporal transformers [31] and incorporation of encoder–decoder architecture [32,33]. In addition, approaches for using transformers for model prediction [34], transformer-based global modeling for multi-object tracking [35], a unified transformer for both single-target and multi-target tracking [36], and an efficient exemplar transformer for real-time application of transformers to visual tracking have been proposed  [37].

While the aforementioned tracking algorithms focus on the problem of short-term tracking where the length of the majority of the test sequences are under a minute, there have been tracking algorithms that focus on tackling the problem of tracking in longer sequences by employing global modeling of the scene around the target. Datasets on the long-term tracking task were also proposed, including LaSOT [14], TLP [38], and OxUvA [15], that consist of longer sequences lasting over a minute with some sequences containing target disappearance and reappearance challenges. Several approaches were proposed where two-stage detector-based GlobalTrack [16] employed the global search strategy and the removal of motion model, with the benefits of re-identifying the target after its disappearance and reappearance, unaffected by the tracker’s previous failures. Siam R-CNN [17] is also based on a two-stage detection framework with a tracklet dynamic programming algorithm. Other approaches. including [26,39]. employ a confidence value-based strategy where global search is performed on a larger search region when the confidence value for target prediction falls under a predefined threshold value. Our proposed framework shares its two-stage detector-based similarity with the aforementioned GlobalTrack [16] but differs in several aspects, including the proposed global context attention module.

Our contributions are as follows: (1) in contrast to GlobalTrack [16] that treats all regions inside the scene *independently* without any context modeling between objects, we introduce a novel global context attention module that can modulate the target feature representation, depending on the context of distractor objects; (2) we obtain higher performance metrics on multiple visual tracking benchmarks using a lighter backbone feature extractor network, ResNet-18 [2], compared to the much heavier backbone network ResNet-50 used in GlobalTrack [16]; and (3) related to (2), we achieve real-time tracking performance of 57 fps, thanks to our proposed global context attention module only contributing to minimal computational load, whereas GlobalTrack [16] shows sub-real-time speeds.

## 3. Proposed Method

**Figure 2:**
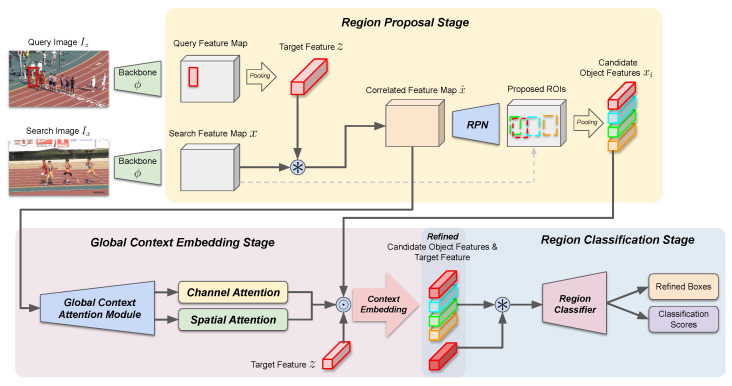
Overview for the proposed tracking framework.

In this section, we describe our proposed global **CO**ntext **A**ttention **T**racker (COAT), where the overall tracking framework largely contains three stages: (1) region proposal stage, (2) global context generation and embedding stage, and (3) the final region classification stage. In the following subsections, we first provide an overview of the proposed tracking framework followed by detailed descriptions on each of the tracking stages, including the proposed global context attention module. Afterwards, we elaborate on the training details and implementation details containing the architectural descriptions and tracking parameter settings. The figure for the overview for our proposed tracking framework is shown in Figure 2. Our tracking framework is composed of a baseline tracker and a global context attention module. The baseline tracker is a two-stage detector-based tracker composed of a region proposal stage and a region classification stage.

### 3.1. Operation of the Baseline Tracker

**Feature extraction:** Given a pair of RGB input frame images $I_{z},\;I_{x}\in R^{H\times W\times 3}$ along with initial bounding box coordinates $b_{1}\in R^{4}$, where $I_{z}$ represents the initial frame (query) image and $I_{x}$ represents the current frame (search) image, respective feature maps are obtained using the shared backbone feature extractor network ϕ(·) where they are denoted as in $\phi \left(I_{z}\right),\;\phi \left(I_{x}\right)\in R^{h\times w\times c}$. The input RGB images have spatial dimensions of height *H* and width *W*, and the feature maps have spatial dimensions of height *h* and width *w* with channel dimension of size *c*. The backbone feature extractor network ϕ(·) is a pretrained CNN from an image classification task [2,21,40], where the final linear classification layer is removed to obtain the intermediate feature representation. In our experiments, we chose the relatively lightweight ResNet-18 [2] for the feature extractor, where we substitute the stride of the final residual block to 1 for increased spatial resolution of the feature map. We also reduce the number of channels by adding 1×1 convolution layers for efficient computation in the following stages. Layers before the final residual block are frozen when training. Additional technical details on the dimensions of the feature maps and training scheme are described in Section 3.3 and Section 3.4.

**Region proposal stage:** After obtaining the query and search feature maps $\phi \left(I_{z}\right),\phi \left(I_{x}\right)\in R^{h\times w\times c}$ using the backbone feature extractor network ϕ(·), a region proposal is performed to find multiple regions that resemble the target object, where these proposed regions serve as candidate regions for subsequent region classification. Using the query feature map $\phi (I_{z})$ and initial bounding box coordinates $b_{1}$, spatially pooled target feature representation $z\in R^{s\times s\times c}$ is obtained using the ROIAlign [41] operation, where *s* is the spatial dimension of the pooled feature representation. Afterwards, the depth-wise cross-correlation operation between the target feature representation *z* and the search feature map $x=\phi (I_{x})$ is performed as in,

(1) $$\hat{x}=x*z,$$

where ∗ denotes the cross-correlation (convolution) operator with unit stride and appropriate zero padding for spatial dimension consistency. The resulting correlated feature map $\hat{x}\in R^{h\times w\times c}$ is then fed into the region proposal network (RPN) module, and a detection head module similar to FCOS [42] is used to find the candidate regions where the centerness prediction branch is removed for simplicity. The RPN module produces two prediction maps using two branches, the classification label map $p\in R^{h\times w\times 2}$ and the bounding box regression map $q\in R^{h\times w\times 4}$ as in,

(2) $$p=f_{cls}\left(\hat{x}\right),\;q=f_{reg}\left(\hat{x}\right),$$

where both branches $f_{cls}$ and $f_{reg}$ include three convolution layers with group normalization [43] and ReLU activation functions in between, respectively. Two prediction maps *p* and *q* have the same spatial dimensions as input feature map $\hat{x}$, where the classification label map *p* contains binary logit values for every position inside the label map, and the bounding box regression map *q* contains coordinate distance values for bounding box estimation. More specifically, for a given position (i,j) in *p*, the corresponding tensor $p_{i,j}\in R^{2}$ represents the binary logits indicating that the position (i,j) is inside or outside the target bounding box. For a position (i,j) in *q*, the corresponding tensor $q_{i,j}\in R^{4}$ represents the distances from (i,j) to the four sides (left, top, right, and bottom) of the estimated bounding box. Subsequently, a softmax operation is conducted on *p*, and a non-maximum suppression (NMS) operation is performed to obtain the top-*N* candidate ROIs.

**Region classification stage:** For candidate ROIs found in the region proposal stage, further region classification is performed to classify the candidate ROIs into positive and negative regions, where a positive label indicates the target region and a negative label indicates the background region. Given top-*N* ROIs with their bounding box coordinates $\left\{r_{1},\ldots ,r_{N}\right\}$ and search feature map *x*, we obtain spatially pooled feature representations $\left\{x_{1},\ldots ,x_{N}\right\}$ by performing ROIAlign operations on *x*, where $x_{i}\in R^{s\times s\times c}$. Subsequently, using the target feature representation *z*, feature modulation is performed for every $x_{i}$ as in,

(3) $${\hat{x}}_{i}=x_{i}⊙z,$$

where ⊙ represents the Hadamard (element-wise) product operation. Afterwards, using the modulated feature representations, region classification and bounding box refinement is performed as in,

(4) $$u_{i}=g_{cls}\left({\hat{x}}_{i}\right),\;v_{i}=g_{reg}\left({\hat{x}}_{i}\right),$$

where both branches $g_{cls}$ and $g_{reg}$ of the region classification network include three convolution layers with group normalization and ReLU activation functions in between, respectively. Output $u_{i}\in R^{2}$ indicates the logits for binary classification between target and background, and $v_{i}={\left[v_{i}^{1},v_{i}^{2},v_{i}^{3},v_{i}^{4}\right]}^{T}\in R^{4}$ represents the regressed values for refining the bounding box coordinates of the ROI $r_{i}$. Lastly, we perform a softmax operation on all of $u_{i}$ to obtain and choose the *k*-th ROI with the highest positive score and perform bounding box refinement for $r_{k}=\left[x_{k}^{c},y_{k}^{c},w_{k},h_{k}\right]$ as in,

(5) $$\begin{array}{c} {x_{k}^{c}}^{\prime }=x_{k}^{c}+w_{k}\cdot v_{k,1},\;\;\;{y_{k}^{c}}^{\prime }=y_{k}^{c}+h_{k}\cdot v_{k,2},\\ w_{k}^{\prime }=w_{k}\cdot \exp\left(v_{k,3}\right),\;\;\;h_{k}^{\prime }=h_{k}\cdot \exp\left(v_{k,4}\right), \end{array}$$

to obtain the refined bounding box $r_{k}^{\prime }=\left[{x_{k}^{c}}^{\prime },{y_{k}^{c}}^{\prime },w_{k}^{\prime },h_{k}^{\prime }\right]$, where $x_{k}^{c}$ and $y_{k}^{c}$ are the center coordinates of the bounding box, and $w_{k}$ and $h_{k}$ are the width and height of the bounding box.

### 3.2. Incorporating the Global Context Attention Module

**Figure 3:**
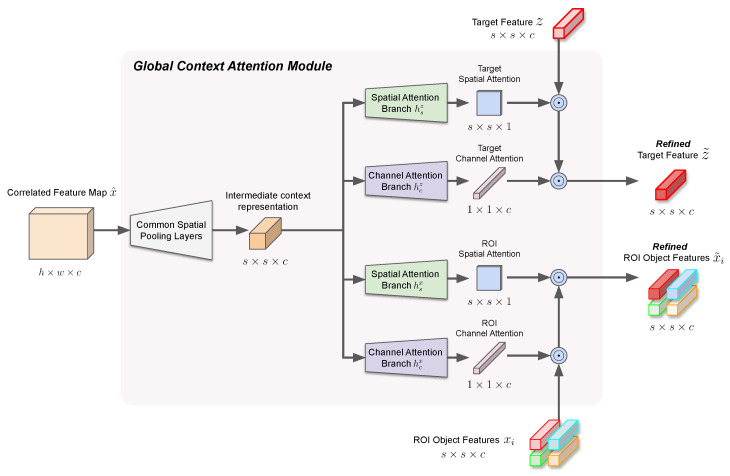
Overview of the global context attention module. Detailed view for the operation of the proposed global context attention module.

Herein, we introduce our proposed module for global context attention and describe the formulation for context aggregation and feature embedding. To obtain the global context information from a given scene, we utilize the correlation map $\hat{x}$ obtained at the region proposal stage. The global context attention module consists of four sub-networks where each operates as in,

(6) $$\begin{array}{c} m_{s}^{x}=h_{s}^{x}\left(\hat{x}\right),\;\;\;m_{s}^{z}=h_{s}^{z}\left(\hat{x}\right),\\ m_{c}^{x}=h_{c}^{x}\left(\hat{x}\right),\;\;\;m_{c}^{z}=h_{c}^{z}\left(\hat{x}\right), \end{array}$$

where global context information is embedded into spatial soft-attention masks $m_{s}^{x},m_{s}^{z}\in R^{s\times s\times 1}$ and channel-wise soft-attention masks $m_{c}^{x},m_{c}^{z}\in R^{1\times 1\times c}$ using four sub-networks of the global context attention module, $h_{s}^{x},h_{s}^{z},h_{c}^{x},h_{c}^{z}$. All sub-networks share the common spatial pooling layers which consist of two convolution layers and adaptive average pooling layer that pools the feature representation into $R^{s\times s\times c}$. After spatial pooling is performed, each sub-network can produce its respective spatial and channel attention masks using two convolution layers and an adaptive average pooling layer, with sigmoid activation at the last layer for restricting the range of the attention masks to [0,1]. Subsequently, spatial and channel attention masks are used in the region classification stage, where attention masks are applied to each spatially pooled ROI features $x_{i}$ and target feature representation *z* as in,

(7) $$\begin{array}{c} {\tilde{x}}_{i}=x_{i}⊙m_{s}^{x}⊙m_{c}^{x},\\ \tilde{z}=z⊙m_{s}^{z}⊙m_{c}^{z}, \end{array}$$

where mismatching dimensions can be broadcasted for shape consistency. After applying the attention masks for both ROI and target feature representations, ROI feature modulation is performed equivalent to Equation (3) as in,

(8) $${\hat{x}}_{i}={\tilde{x}}_{i}⊙\tilde{z},$$

where subsequent region classification and bounding box refinement branches are equivalent to Equation (4). By applying two separate attention masks to ROI and the target feature representations, we can increase the adaptability of our proposed module to various scenes. An overview for the structure and operation of our proposed global context attention module is shown in Figure 3, and the overall step-by-step operation of our proposed tracking algorithm is shown in Algorithm 1.
**Algorithm 1:** Visual tracking with global context attention
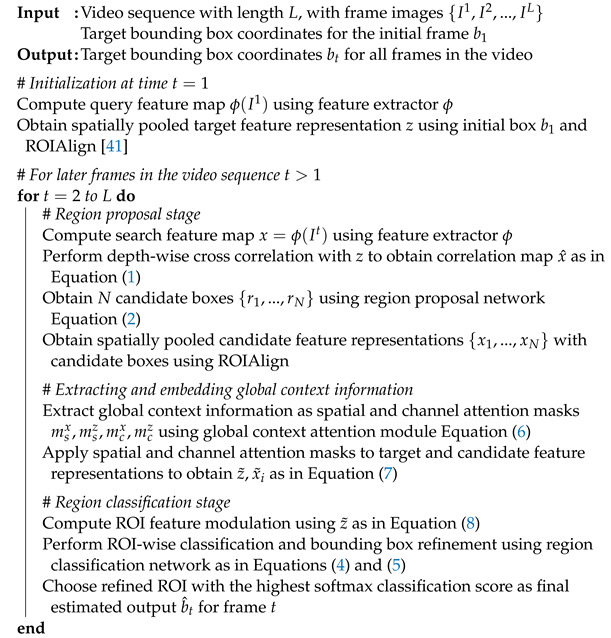


### 3.3. Training the Overall Tracking Framework

In order to train the overall tracking framework, we enforce two separate loss functions for the region proposal network and the region classification network. The global context attention module is trained with upstream gradients delivered from enforcing the loss function on the region classification network using backpropagation.

**Loss function for the region proposal network:** To train the region proposal network, we enforce loss functions that are similar to the ones used in the object detection framework FCOS [42] where we train two branches, the binary classification branch $f_{cls}$ and the bounding box regression branch $f_{reg}$. Total RPN loss is denoted as $L_{RPN}^{f}$, loss for the classification branch is denoted as $L_{cls}^{f}$, and loss for the regression branch is denoted as $L_{reg}^{f}$ with respective loss functions formulated as follows,

(9) $$L_{RPN}^{f}\left(\left\{p,p^{*}\right\},\left\{q,q^{*}\right\}\right)=\frac{1}{hw}\sum_{i,j}L_{cls}^{f}\left(p_{i,j},p_{i,j}^{*}\right)+\frac{\lambda_{RPN}}{N_{pos}}1_{\left\{p_{i,j}^{*}>0\right\}}\sum_{i,j}L_{reg}^{f}\left(q_{i,j},q_{i,j}^{*}\right),$$

where $p\in R^{h\times w\times 2}$ and $\;q\in R^{h\times w\times 4}$ are the output maps of the classification and regression branches, respectively, and $p^{*}$ and $q^{*}$ are the ground truth label maps with the identical spatial dimensions. $N_{pos}$ is the number of positions in $p^{*}$ with positive labels. For the classification branch, focal loss [44] $L_{cls}^{f}$ is enforced for every position (i,j) inside the output classification map *p*, where $p_{i,j}^{*}=1$ for positions inside the bounding box of the target object, and $p_{i,j}^{*}=0$ for the remaining positions in the background region with the following formulation,

(10) $$L_{cls}^{f}\left(p_{i,j},p_{i,j}^{*}\right)=-p_{i,j}^{*}{\left(1-p_{i,j}\right)}^{\gamma }\log\left(p_{i,j}\right)-\left(1-p_{i,j}^{*}\right)p_{i,j}^{\gamma }\log\left(1-p_{i,j}\right),$$

where γ is the focusing parameter that controls the weighting for easy/hard examples. For the regression branch, IoU loss, which is based on the L1 distance, is enforced only on the positions inside the target bounding box ($p_{i,j}^{*}>0$) with the following formulation,

(11) $$L_{reg}^{f}\left(q_{i,j},q_{i,j}^{*}\right)=1-\frac{|q_{i,j}⋂q_{i,j}^{*}|}{|q_{i,j}⋃q_{i,j}^{*}|},$$

where |·| denotes the area of the region, ⋂ is the intersection between two bounding boxes, and ⋃ is the union over two bounding boxes.

**Loss function for the region classification network:** At the training stage, a region classification network is trained with loss functions that are similar to the ones used for the RPN. Loss is enforced on the region classification branch $g_{cls}$ and the bounding box refinement branch $g_{reg}$, where total loss is denoted as $L_{RCL}^{g}$, the loss term for the classification branch is denoted as $L_{cls}^{g}$, and the loss term for the bounding box refinement branch is denoted as $L_{reg}^{g}$ with respective loss functions formulated as follows,

(12) $$L_{RCL}^{g}\left(\left\{u,u^{*}\right\},\left\{v,v^{*}\right\}\right)=\frac{1}{N}\sum_{i}L_{cls}^{g}\left(u_{i},u_{i}^{*}\right)+\frac{\lambda_{RCL}}{N_{pos}}1_{\left\{u_{i}^{*}>0\right\}}\sum_{i}L_{reg}^{g}\left(v_{i},v^{*}\right),$$

where $u\in R^{N\times 2}$ and $v\in R^{N\times 4}$ are the output logits and refined bounding box coordinates, respectively, $u^{*}\in R^{N}$ and $v^{*}\in R^{4}$ are the ground truth labels for each output branch, and $N_{pos}$ is the number of output boxes with positive class labels. For the classification branch, loss is enforced on all *N* candidate ROI bounding boxes where the ground truth class label $u_{i}^{*}$ for the candidate box is determined by the IoU value $\mathrm{IoU}(r_{i},v^{*})$ calculated between the candidate box $r_{i}$ and the target bounding box $v^{*}$, where $u_{i}^{*}=1$ is assigned if $\mathrm{IoU}\left(r_{i},v^{*}\right)>\tau_{p}$ and $u_{i}^{*}=0$ if $\mathrm{IoU}\left(r_{i},v^{*}\right)<\tau_{n}$. In our implementation, we use threshold values $\left(\tau_{p},\tau_{n}\right)=\left(0.5,0.4\right)$, and the loss is not enforced on candidate boxes without labels. For the classification branch loss $L_{cls}^{g}\left(u_{i},u_{i}^{*}\right)$, the same focal loss used in training the classification branch of RPN is used as in Equation (10). In addition, for the regression branch loss $L_{reg}^{g}\left(v_{i},v^{*}\right)$, the same IoU loss used in training the regression branch of RPN is used as in Equation (11).

**Optimizing the overall loss function:** Using the individual loss functions defined above, we formulate the overall loss function $L_{total}$ as in:

(13) $$L_{total}\left(\left\{p,p^{*}\right\},\left\{q,q^{*}\right\},\left\{u,u^{*}\right\},\left\{v,v^{*}\right\}\right)=L_{RPN}^{f}\left(\left\{p,p^{*}\right\},\left\{q,q^{*}\right\}\right)+\lambda L_{RCL}^{g}\left(\left\{u,u^{*}\right\},\left\{v,v^{*}\right\}\right),$$

where λ is used for balancing the importance between two loss functions. To stabilize the overall training process, we start from λ=0 at the beginning of the training process, only training the RPN. When a sufficient number of training iterations is achieved (e.g., we used $10^{4}$ iterations in our experiments), we change the value to λ=1 to start jointly training the RPN, the region classification network, and the global context attention module. This prevents the training of the region classification network when the majority of the region proposals made from the RPN are labeled negative at the beginning of the training stage, where they can enforce a strong negative bias on the region classification network and the global context attention module. By minimizing the overall loss function above using a gradient descent-based optimization algorithm, the RPN and the region classification network can be trained simultaneously, while the proposed global context attention module can be trained using the upstream gradients from the region classification network.

### 3.4. Implementation Details

Herein, we clarify the comprehensive details on the proposed architecture, parameter settings, training dataset used, and training details.

#### 3.4.1. Architectural Details and Parameter Settings

For the backbone feature extractor network ϕ(·), we employ ResNet-18 [2] since it is relatively lightweight compared to other backbone feature extractors used in other tracking algorithms [16,30,31], where it retains the real-time speed while demonstrating adequate performance. All input frame images are resized to H=400,W=666 before they can be processed by the tracking framework, where the original aspect ratio is retained by matching the length of the longest edge to the corresponding *H* or *W* and adding zero-padding to the bottom or right side of the image. The feature map produced by ϕ(·) has the spatial dimension of h=25,w=42, where a 1×1 convolution layer is added at the final stage to reduce the size of the channel dimension from the original $c^{\prime }=512$ to c=256.

The region proposal network and the region classification network operate on the same correlated feature map $\hat{x}\in R^{h\times w\times c}$ where the RPN spatially pools the target feature representation *z* into a size of s=5. Both branches $f_{cls}$ and $f_{reg}$ have two 1×1 convolution layers with input and output channel sizes of c=256, where group normalization [43] with the number of groups G=16 and ReLU activation are inserted in between. The final 3×3 convolution layer is used to convert the feature maps to appropriate dimensions of *p* and *q*. As output of the RPN, the total of the top N=64 ROI candidate boxes are obtained using non-maximum suppression with an overlap threshold value of 0.9. Using the ROI boxes $r_{i}$, the region classification network spatially pools from *x* to obtain candidate feature representations $x_{i}$ with a size of s=5. Both branches $g_{cls}$ and $g_{reg}$ are formulated identically to $f_{cls}$ and $f_{reg}$ except for the final convolution layer of filter size s×s, where it maps the feature representation into binary logits and regressed bounding box values, respectively.

The global context attention module operates on the correlated feature map $\hat{x}$ and contains four network branches $h_{s}^{x},h_{s}^{z},h_{c}^{x},h_{c}^{z}$, where $h_{s}^{x}$ and $h_{s}^{z}$ are used to produce spatial masks, and $h_{c}^{x}$ and $h_{c}^{z}$ are used to produce channel masks, respectively. All branches share common layers composed of two 3×3 convolution layers with group normalization and ReLU in between, and subsequent adaptive spatial pooling layer pools the feature map into a 7×7 feature map to summarize the scene-specific context information. Then, spatial mask branches $h_{s}^{x}$ and $h_{s}^{z}$ process the feature map using two 3×3 convolution layers with group normalization and ReLU in between, followed by spatial adaptive pooling to s×s and sigmoid activation. Similarly, channel mask branches $h_{c}^{x}$ and $h_{c}^{z}$ also contain two 3×3 convolution layers with group normalization and ReLU in between, followed by spatial adaptive pooling to 1×1 and sigmoid activation.

#### 3.4.2. Training Data

To train the overall framework, training splits of ImageNetVID [45], LaSOT [14], GOT-10k [46], and YouTubeBB [47] datasets are used, with a subset of the LaSOT dataset assigned for the validation set. From these large-scale video datasets, pairs of query images $I_{z}$ and search images $I_{x}$ are sampled from a randomly chosen video sequence along with their bounding box annotations, where a specific dataset is first randomly chosen based on the probability proportional to its size. For sampled image pairs, random data augmentations, each with probability of 0.2, i.i.d., are performed for improved generalization, including horizontal flips, additive Gaussian noise, color jittering, and Gaussian blurring. In addition, bounding box coordinates are randomly jittered by ±1% of the original box height/width. Pixel intensity values for the augmented input images are first scaled to [0,1], then RGB channels for all input images are normalized as in $\frac{I-\mu }{\sigma }$, using the mean μ and standard deviation σ values from the default setting of the original ResNet [2], where μ=[0.485,0.456,0.406] and σ=[0.229,0.224,0.225] are used in practice.

#### 3.4.3. Training Details and Hyperparameters

To optimize the loss function in Equation (13), we used the Adam [48] optimizer with a batch size of 16 image pairs per each training iteration. The learning rate was set to $10^{-4}$, and the weight decay penalty was set to $10^{-5}$. Training was performed for 10 epochs with each epoch of $10^{6}$ iterations, and the learning rate was decayed by a factor of 0.9 for every epoch. For initialization, we employed the pretrained weights from the ResNet-18 architecture, and we froze the weights except for the last residual block where its stride was modified to 1 for increased resolution of the output feature map. For the focal loss terms used for training the classification branches $f_{cls},g_{cls}$ as defined in Equation (10), we use a focusing hyperparameter value of γ=2.0 in our experiments. For the balancing terms in Equations (9) and (12), $\lambda_{RPN}=\lambda_{RCL}=1$ was used for simplicity. We implement our framework using Python 3.6 and PyTorch [49] library (v1.6.0) on Ubuntu 16.04. For the hardware environment, we run our algorithm and perform run-time speed measurements on a single Nvidia Tesla V100 GPU with 32GB of VRAM with Intel Xeon CPU and 128GB of RAM.

## 4. Experiments

In this section, to evaluate our proposed tracking algorithm, we use large-scale tracking benchmarks LaSOT [14] and GOT-10k [46] for our experiments, representative of long-term and short-term tracking tasks, respectively. We perform quantitative and qualitative evaluations to compare our proposed tracking algorithm with other recently proposed tracking algorithms. In addition, we perform ablation experiments on our proposed global context attention module to further validate the performance gains and show the effectiveness of the proposed global context attention module.

### 4.1. Quantitative Evaluation

For the quantitative evaluations, we evaluate our tracker on the test sets of LaSOT [14] and GOT-10k [46], where we fix all parameters for all benchmarks and experiments. LaSOT is a large-scale, long-term tracking benchmark where average length of all sequences is longer than one minute, whereas GOT-10k has shorter sequences but includes a larger number of sequences with more diverse categories of target objects.

The LaSOT [14] dataset is a large-scale, long-term tracking video dataset containing 1400 sequences, where the average sequence length is 2512 frames (83.7 s under 30 fps setting), with a total of approximately 3.5 M annotated frames. All frames are annotated with bounding box coordinates of the target object, with occlusion and out-of-view frame indications, and sequence-level natural language descriptions of the target object. We evaluate our proposed tracking algorithm on the test split of the LaSOT dataset, which contains 280 sequences. We evaluate our tracker based on the performance metrics of area-under-curve (AUC) of the success plots, location precision plots, and normalized precision plots. The GOT-10k [46] dataset is a large-scale, short-term oriented tracking dataset containing 10,000 sequences in total, where its test split contains 420 sequences for evaluation with average sequence length of 150 frames (15 s under 10 fps setting). The dataset is collected to evaluate the one-shot generalization performance of the tracking algorithm where the training split and test split have disjoint object categories. Evaluation metrics include calculating the success rate (SR) with two overlap threshold values 0.5 and 0.75 and an average overlap (AO) value of the estimated bounding box and the ground truth bounding box. All evaluations were performed under the conventional OPE (one-pass evaluation) setting where the tracker is initialized using the ground truth bounding box given at the first frame and run sequentially throughout the subsequent test sequence. The tracker produces the output of estimated bounding boxes for each frame, where they are evaluated using precision and overlap values.

**Figure 4:**
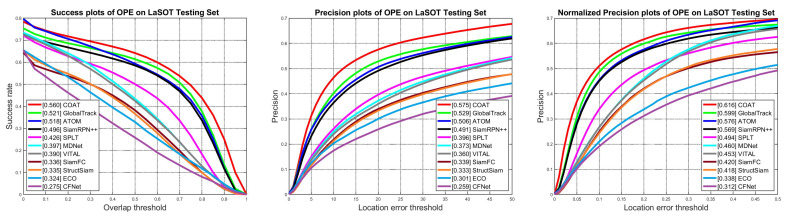
Success and precision plots for LaSOT test set. Best viewed on high-resolution display.

**Comparison with other trackers:** We evaluated our proposed tracking algorithm on the LaSOT test set and provide the results in Table 1. We denote our tracker, global **CO**ntext **A**ttention **T**racker as **COAT**, in the subsequent tables and figures. For comparison, we mainly chose trackers with similar backbone feature extractor architectures, where GlobalTrack [16], ATOM [50], DiMP [51], SPLT [52], SiamRPN++ [4], and Ocean [53] have ResNet [2] as backbone and are chosen for comparison. In this manner, we can provide a fair comparison in terms of model complexity and feature representation. The results show that our proposed tracking algorithm outperforms other ResNet backbone-based tracking algorithms, which are SiamRPN++ [4], ATOM [50], SPLT [52], and our baseline tracking algorithm GlobalTrack [16]. Additionally, our tracker runs faster than most tracking algorithms, running at a real-time speed of 57 fps, whereas the baseline GlobalTrack runs at sub real-time speed. We obtained higher performance on all metrics compared to GlobalTrack using the lighter backbone network ResNet-18, whereas GlobalTrack employs ResNet-50, achieving comparable performance to DiMP-50 which also employs ResNet-50 as backbone. Detailed success and precision plots with varying threshold values of AUC, precision, and normalized precision are also shown in Figure 4.

Furthermore, we also evaluated our tracker on the GOT-10k test set and provide the result in Table 2, where we show the performance on relatively short-term tracking scenarios. COAT achieves competitive performance in terms of success rate and average overlap metrics, outperforming most tracking algorithms that were oriented for short-term tracking tasks. Performance metrics were obtained using the online evaluation site provided by the authors. Even without any temporal smoothness priors, motion priors, and meticulous tuning of cosine window weighting, COAT manages to achieve competitive performance with the same parameter settings used for long-term tracking, showing its generalizability on a wide range of visual tracking tasks.

### 4.2. Ablation Experiments

To perform more in-depth analysis on COAT and the proposed global context attention module, we performed additional ablation analysis on the challenge attributes and component-wise comparisons. For the ablation experiments, AUC of the success plot obtained on the LaSOT test set was used for the evaluation metric.

**Figure 5:**
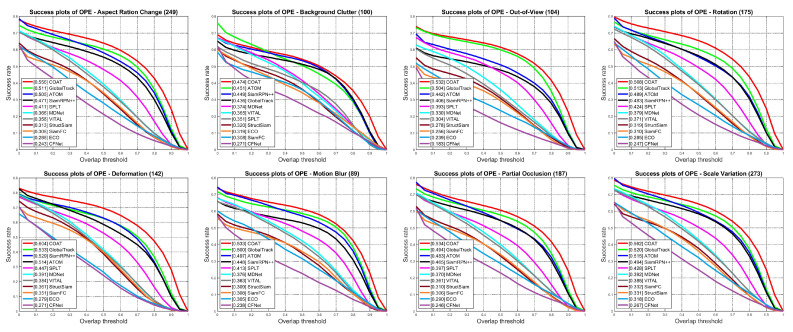
Success plots for eight challenge attributes of LaSOT test set. Best viewed on high-resolution display.

**Attribute-wise ablation:** To analyze the effectiveness of our proposed global context attention module with COAT, we show the success plots under eight different challenge attributes of the LaSOT test set in Figure 5. COAT was able to achieve high performance metrics on all eight challenge attributes, where it achieves noteworthy performance gains on attributes of aspect ratio change, deformation, rotation, scale variation, and background clutter compared to the baseline tracker GlobalTrack, owing to the effect of context information provided by the proposed global context attention module. Using our global context attention module, our proposed tracker is able to fully utilize the context information from the given scene to accurately localize the target under various challenging circumstances. Additionally, the full-frame search-based GlobalTrack shows lower performance on the background clutter attribute compared to ATOM and SiamRPN++, due to the frequent errors made by mislabeling similar background object as the target. Even with the same full-frame search-based design, our proposed COAT is able to successfully suppress the errors originating from the background clutter and achieve high performance metrics.

**Component-wise ablation:** To precisely quantify the effectiveness of our propose global context attention module, we report the performance metrics on the LaSOT test set without the proposed module in Table 3.

From the results, we can validate the effectiveness of our proposed module where it achieves +2.4% performance gain on the AUC metric. When compared with GlobalTrack, our tracker without the proposed module also achieves performance gains, where this is speculated to be due to the changes in design of the region proposal network. The anchor-free design [42] of the region proposal network can localize candidate regions of various scales and aspect ratios compared to previous network design that relies on the predesignated template anchor boxes. In addition, the anchor-free design reduces the number of parameters compared to previous anchor-box-based design, where it is less prone to overfitting to certain sizes and shapes of candidate regions.

### 4.3. Qualitative Evaluation

In this section, we visualize the qualitative outputs of our proposed tracking algorithm on the LaSOT test set to facilitate further understanding of the characteristics of our proposed tracking algorithm. We first show the comparison between output bounding boxes produced by different tracking algorithms and show some sample visualization of the attention weights to provide an explanation for the role of the attention weights when locating the target object.

**Figure 6:**
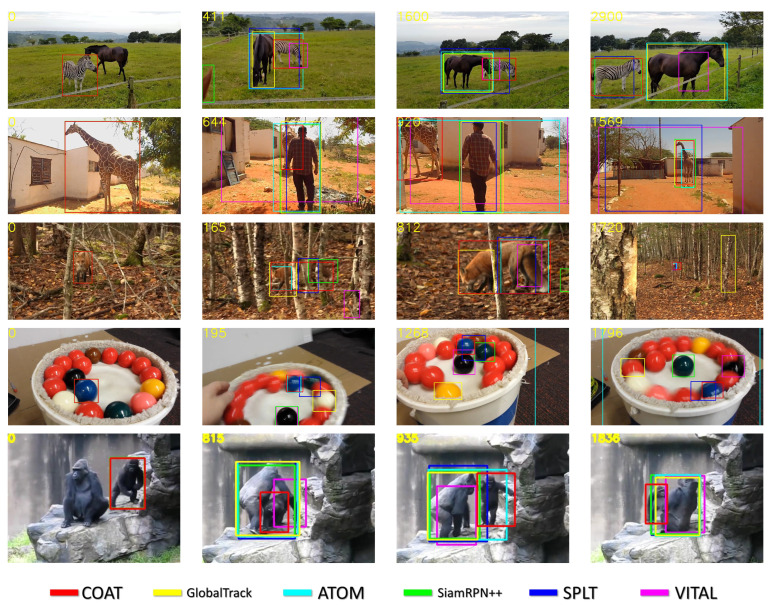
Qualitative results on the LaSOT test set. Tracking results are for sequences *zebra-17, giraffe-10, fox-5, pool-12, and gorilla-9*. Best viewed on a high resolution display.

**Qualitative comparison:** We present qualitative results and compare our proposed tracker to GlobalTrack, ATOM, SiamRPN++, SPLT, and VITAL in Figure 6 for selected sequences from the LaSOT test set. The color of the bounding box denotes the result of a specific tracker, and frame indices are shown in the top-left corner. For sequences *zebra-17*, *pool-12*, and *gorilla-9*, where objects of similar class and appearance simultaneously appear in a scene, our tracker can successfully locate the correct target, whereas other trackers fail to discriminate similar objects. In addition, our tracker can successfully handle scenarios with partial occlusion such as in *fox-5*, and can recover from out-of-scene disappearances where a target completely leaves the scene, such as in *giraffe-10*, whereas other trackers fail to find the correct region of the target object. Strengths of our tracker for the aforementioned attributes were also quantified in the earlier attribute-wise ablation experiments, where success plots for background clutter, partial occlusion, and out-of-view attributes are visualized in Figure 5.

**Figure 7:**
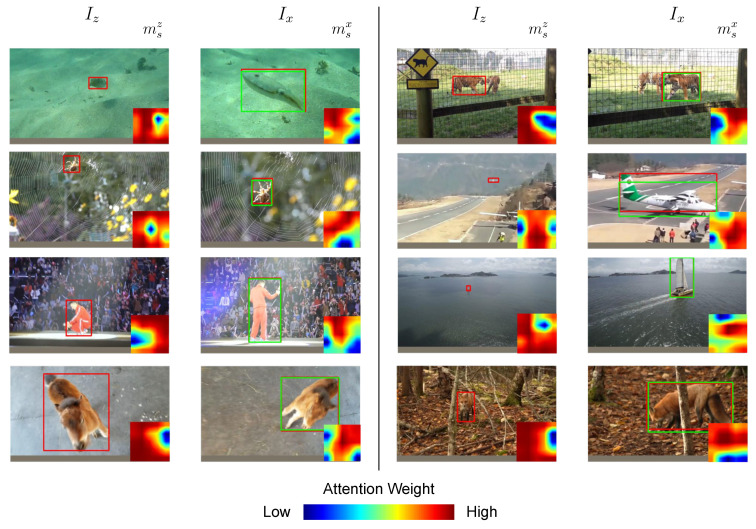
Visualization of spatial attention masks on the LaSOT test set.

**Visualizing the attention weights:** We present sample visualizations of the spatial attention masks in Figure 7. We show query and search images, $I_{z}$ and $I_{x}$, where they are annotated with ground truth bounding boxes (red) and the estimated bounding boxes (green). Spatial attention weights $m_{s}^{z}$ and $m_{s}^{x}$ are visualized in the bottom-right corner of respective images, where relative magnitudes of the attention weights are color-coded according to the map in the bottom of the figure. For the query image $I_{z}$ and search image $I_{x}$, where target feature *z* and candidate features $x_{i}$ are obtained using the backbone network and the region proposal network, spatial attention weights $m_{s}^{z}$ and $m_{s}^{x}$ obtained from the global context attention module are applied to the respective features as in Equation (7). We visualize the relative magnitude of the attention weights using color-coded heatmaps, where parts with higher attention weights are considered more significant and helpful when discriminating the target in the subsequent region classification stage, and vice versa for parts with lower attention weights. We can see that the spatial attention weights can adaptively change according to the target object and its context, where the global context attention module is learned to focus on different object parts for discriminating and correctly identifying the target object.

## 5. Conclusions

In this paper, we proposed a novel global context attention module for visual tracking, where our proposed module can extract and summarize the global scene information to provide the target feature representation with spatial and channel attention weights. The context-modulated target feature representation can be used to discriminate similar objects using relevant feature channels and spatial parts. We show the effectiveness of our proposed method on two large-scale visual tracking benchmarks, test splits of LaSOT and GOT-10k, where our tracker achieves competitive performance metrics on both datasets, while running at a real-time speed. Our tracker shows large improvements in both performance and speed compared to the baseline GlobalTrack, with higher metrics on all challenge attributes of the LaSOT benchmark. Additional ablation experiments also validate the effectiveness of our proposed module for various challenging attributes of visual tracking, achieving improved performance on numerous challenging scenarios owing to the success of the proposed global context embedding module.

## Figures and Tables

**Table 1:** Quantitative comparison on the **LaSOT** test set.

	COAT	GlobalTrack [16]	ATOM [50]	DiMP-50 [51]	SiamRPN++ [4]	DASiam [26]	SPLT [52]	MDNet [19]	Ocean [53]	SiamFC [3]	CFNet [54]
AUC	0.556	0.521	0.518	0.569	0.496	0.448	0.426	0.397	0.560	0.336	0.275
Precision	0.575	0.529	0.506	-	0.491	0.427	0.396	0.373	0.566	0.339	0.259
Norm. Precision	0.616	0.599	0.576	0.650	0.569	-	0.494	0.460	-	0.420	0.312
FPS	57	6	30	43	35	110	25.7	0.9	25	58	43

**Table 2:** Quantitative comparison on the **GOT-10k** test set.

(%)	COAT	ATOM [50]	DiMP-50 [51]	SiamMask [11]	Ocean [53]	CFNet [54]	SiamFC [3]	GOTURN [55]	CCOT [22]	ECO [23]	CF2 [56]	MDNet [19]
${\mathrm{SR}}_{0.50}$	64.3	63.4	71.7	58.7	72.1	40.4	35.3	37.5	32.8	30.9	29.7	30.3
${\mathrm{SR}}_{0.75}$	49.1	40.2	49.2	36.6	-	14.4	9.8	12.4	10.7	11.1	8.8	9.9
AO	57.2	55.6	61.1	51.4	61.1	37.4	34.8	34.7	32.5	31.6	31.5	29.9

**Table 3:** Ablation analysis on the global context attention module. Results without the global context attention module are shown in the second column.

	COAT	COAT *w/o* GCAM	GlobalTrack [16]
AUC	0.556	0.532	0.521
Precision	0.575	0.541	0.529
Norm. Precision	0.616	0.605	0.599

## Data Availability

The datasets used in the study are available from the corresponding authors upon reasonable request.

## References

[1] Krizhevsky A., Sutskever I., Hinton G.E. ImageNet classification with deep convolutional neural networks. Proceedings of the NIPS.

[2] He K., Zhang X., Ren S., Sun J. Deep residual learning for image recognition. Proceedings of the CVPR.

[3] Bertinetto L., Valmadre J., Henriques J.F., Vedaldi A., Torr P.H. (2016). Fully-Convolutional Siamese Networks for Object Tracking. arXiv.

[4] Li B., Wu W., Wang Q., Zhang F., Xing J., Yan J. Siamrpn++: Evolution of siamese visual tracking with very deep networks. Proceedings of the CVPR.

[5] Zhang Z., Peng H. Deeper and wider siamese networks for real-time visual tracking. Proceedings of the CVPR.

[6] Ren S., He K., Girshick R., Sun J. Faster R-CNN: Towards real-time object detection with region proposal networks. Proceedings of the NIPS.

[7] Li B., Yan J., Wu W., Zhu Z., Hu X. High Performance Visual Tracking With Siamese Region Proposal Network. Proceedings of the CVPR.

[8] Ma H., Acton S.T., Lin Z. (2022). CAT: Centerness-Aware Anchor-Free Tracker. Sensors.

[9] Chen Z., Zhong B., Li G., Zhang S., Ji R. Siamese Box Adaptive Network for Visual Tracking. Proceedings of the CVPR.

[10] Xu Y., Wang Z., Li Z., Yuan Y., Yu G. SiamFC++: Towards robust and accurate visual tracking with target estimation guidelines. Proceedings of the AAAI.

[11] Wang Q., Zhang L., Bertinetto L., Hu W., Torr P.H. Fast online object tracking and segmentation: A unifying approach. Proceedings of the CVPR.

[12] Wu Y., Lim J., Yang M.H. (2015). Object tracking benchmark. IEEE TPAMI.

[13] Čehovin L., Leonardis A., Kristan M. (2016). Visual object tracking performance measures revisited. IEEE TIP.

[14] Fan H., Lin L., Yang F., Chu P., Deng G., Yu S., Bai H., Xu Y., Liao C., Ling H. Lasot: A high-quality benchmark for large-scale single object tracking. Proceedings of the CVPR.

[15] Valmadre J., Bertinetto L., Henriques J.F., Tao R., Vedaldi A., Smeulders A.W., Torr P.H., Gavves E. Long-term tracking in the wild: A benchmark. Proceedings of the ECCV.

[16] Huang L., Zhao X., Huang K. GlobalTrack: A Simple and Strong Baseline for Long-term Tracking. Proceedings of the AAAI.

[17] Voigtlaender P., Luiten J., Torr P.H., Leibe B. Siam r-cnn: Visual tracking by re-detection. Proceedings of the CVPR.

[18] Wang N., Yeung D.Y. Learning a deep compact image representation for visual tracking. Proceedings of the NIPS.

[19] Nam H., Han B. Learning Multi-Domain Convolutional Neural Networks for Visual Tracking. Proceedings of the CVPR.

[20] Jung I., Son J., Baek M., Han B. Real-time mdnet. Proceedings of the ECCV.

[21] Simonyan K., Zisserman A. (2014). Very deep convolutional networks for large-scale image recognition. arXiv.

[22] Danelljan M., Robinson A., Khan F.S., Felsberg M. Beyond correlation filters: Learning continuous convolution operators for visual tracking. Proceedings of the ECCV.

[23] Danelljan M., Bhat G., Khan F., Felsberg M. ECO: Efficient Convolution Operators for Tracking. Proceedings of the CVPR.

[24] Bhat G., Johnander J., Danelljan M., Shahbaz Khan F., Felsberg M. Unveiling the power of deep tracking. Proceedings of the ECCV.

[25] Xu T., Feng Z.H., Wu X.J., Kittler J. Joint group feature selection and discriminative filter learning for robust visual object tracking. Proceedings of the ICCV.

[26] Zhu Z., Wang Q., Li B., Wu W., Yan J., Hu W. Distractor-Aware Siamese Networks for Visual Object Tracking. Proceedings of the ECCV.

[27] Cheng L., Zheng X., Zhao M., Dou R., Yu S., Wu N., Liu L. (2022). SiamMixer: A Lightweight and Hardware-Friendly Visual Object-Tracking Network. Sensors.

[28] Yan B., Peng H., Wu K., Wang D., Fu J., Lu H. LightTrack: Finding Lightweight Neural Networks for Object Tracking via One-Shot Architecture Search. Proceedings of the CVPR.

[29] Vaswani A., Shazeer N., Parmar N., Uszkoreit J., Jones L., Gomez A.N., Kaiser Ł., Polosukhin I. Attention is all you need. Proceedings of the NIPS.

[30] Chen X., Yan B., Zhu J., Wang D., Yang X., Lu H. Transformer Tracking. Proceedings of the CVPR.

[31] Yan B., Peng H., Fu J., Wang D., Lu H. Learning Spatio-Temporal Transformer for Visual Tracking. Proceedings of the ICCV.

[32] Yu B., Tang M., Zheng L., Zhu G., Wang J., Feng H., Feng X., Lu H. High-Performance Discriminative Tracking With Transformers. Proceedings of the ICCV.

[33] Yang C., Zhang X., Song Z. (2022). CTT: CNN Meets Transformer for Tracking. Sensors.

[34] Mayer C., Danelljan M., Bhat G., Paul M., Paudel D.P., Yu F., Van Gool L. Transforming Model Prediction for Tracking. Proceedings of the CVPR.

[35] Zhou X., Yin T., Koltun V., Krähenbühl P. Global Tracking Transformers. Proceedings of the CVPR.

[36] Ma F., Shou M.Z., Zhu L., Fan H., Xu Y., Yang Y., Yan Z. Unified Transformer Tracker for Object Tracking. Proceedings of the CVPR.

[37] Blatter P., Kanakis M., Danelljan M., Van Gool L. Efficient Visual Tracking With Exemplar Transformers. Proceedings of the WACV.

[38] Moudgil A., Gandhi V. Long-term Visual Object Tracking Benchmark. Proceedings of the ACCV.

[39] Dai K., Zhang Y., Wang D., Li J., Lu H., Yang X. High-performance long-term tracking with meta-updater. Proceedings of the CVPR.

[40] Szegedy C., Liu W., Jia Y., Sermanet P., Reed S., Anguelov D., Erhan D., Vanhoucke V., Rabinovich A. Going deeper with convolutions. Proceedings of the CVPR.

[41] He K., Gkioxari G., Dollár P., Girshick R. Mask r-cnn. Proceedings of the ICCV.

[42] Tian Z., Shen C., Chen H., He T. FCOS: Fully convolutional one-stage object detection. Proceedings of the ICCV.

[43] Wu Y., He K. Group normalization. Proceedings of the ECCV.

[44] Lin T.Y., Goyal P., Girshick R., He K., Dollár P. Focal loss for dense object detection. Proceedings of the ICCV.

[45] Russakovsky O., Deng J., Su H., Krause J., Satheesh S., Ma S., Huang Z., Karpathy A., Khosla A., Bernstein M. (2015). Imagenet large scale visual recognition challenge. IJCV.

[46] Huang L., Zhao X., Huang K. (2019). GOT-10k: A Large High-Diversity Benchmark for Generic Object Tracking in the Wild. arXiv.

[47] Real E., Shlens J., Mazzocchi S., Pan X., Vanhoucke V. Youtube-boundingboxes: A large high-precision human-annotated data set for object detection in video. Proceedings of the CVPR.

[48] Kingma D., Ba J. (2015). Adam: A method for stochastic optimization. arXiv.

[49] Paszke A., Gross S., Massa F., Lerer A., Bradbury J., Chanan G., Killeen T., Lin Z., Gimelshein N., Antiga L. Pytorch: An imperative style, high-performance deep learning library. Proceedings of the NeurIPS.

[50] Danelljan M., Bhat G., Khan F.S., Felsberg M. Atom: Accurate tracking by overlap maximization. Proceedings of the CVPR.

[51] Bhat G., Danelljan M., Gool L.V., Timofte R. Learning discriminative model prediction for tracking. Proceedings of the ICCV.

[52] Yan B., Zhao H., Wang D., Lu H., Yang X. ‘Skimming-Perusal’Tracking: A Framework for Real-Time and Robust Long-term Tracking. Proceedings of the ICCV.

[53] Zhang Z., Peng H., Fu J., Li B., Hu W. Ocean: Object-aware Anchor-free Tracking. Proceedings of the ECCV.

[54] Valmadre J., Bertinetto L., Henriques J., Vedaldi A., Torr P.H.S. End-To-End Representation Learning for Correlation Filter Based Tracking. Proceedings of the CVPR.

[55] Held D., Thrun S., Savarese S. Learning to track at 100 fps with deep regression networks. Proceedings of the ECCV.

[56] Ma C., Huang J.B., Yang X., Yang M.H. Hierarchical convolutional features for visual tracking. Proceedings of the ICCV.

